# DaTeR: error-correcting phylogenetic chronograms using relative time constraints

**DOI:** 10.1093/bioinformatics/btad084

**Published:** 2023-02-08

**Authors:** Abhijit Mondal, L Thiberio Rangel, Jack G Payette, Gregory P Fournier, Mukul S Bansal

**Affiliations:** Department of Computer Science and Engineering, University of Connecticut, Storrs, CT 06269, USA; Department of Earth, Atmospheric and Planetary Sciences, Massachusetts Institute of Technology, Cambridge, MA 02139, USA; Department of Earth, Atmospheric and Planetary Sciences, Massachusetts Institute of Technology, Cambridge, MA 02139, USA; Department of Earth, Atmospheric and Planetary Sciences, Massachusetts Institute of Technology, Cambridge, MA 02139, USA; Department of Computer Science and Engineering, University of Connecticut, Storrs, CT 06269, USA; Institute for Systems Genomics, University of Connecticut, Storrs, CT 06269, USA

## Abstract

**Motivation:**

A *chronogram* is a dated phylogenetic tree whose branch lengths have been scaled to represent time. Such chronograms are computed based on available date estimates (e.g. from dated fossils), which provide absolute time constraints for one or more nodes of an input undated phylogeny, coupled with an appropriate underlying model for evolutionary rates variation along the branches of the phylogeny. However, traditional methods for phylogenetic dating cannot take into account *relative* time constraints, such as those provided by inferred horizontal transfer events. In many cases, chronograms computed using only absolute time constraints are inconsistent with known relative time constraints.

**Results:**

In this work, we introduce a new approach, Dating Trees using Relative constraints (*DaTeR*), for phylogenetic dating that can take into account both absolute and relative time constraints. The key idea is to use existing Bayesian approaches for phylogenetic dating to sample posterior chronograms satisfying desired absolute time constraints, minimally adjust or ‘error-correct’ these sampled chronograms to satisfy all given relative time constraints, and aggregate across all error-corrected chronograms. DaTeR uses a constrained optimization framework for the error-correction step, finding minimal deviations from previously assigned dates or branch lengths. We applied DaTeR to a biological dataset of 170 Cyanobacterial taxa and a reliable set of 24 transfer-based relative constraints, under six different molecular dating models. Our extensive analysis of this dataset demonstrates that DaTeR is both highly effective and scalable and that its application can significantly improve estimated chronograms.

**Availability and implementation:**

Freely available from https://compbio.engr.uconn.edu/software/dater/

**Supplementary information:**

Supplementary data are available at *Bioinformatics* online.

## 1 Introduction

The inference of dated phylogenies, also referred to as *time trees* or *chronograms*, is an important problem with many applications in evolutionary biology. Given an undated phylogenetic tree, with branch lengths expressed in terms of the average number of substitutions per site, along with estimated dates for a subset of nodes on the phylogeny, the phylogenetic dating problem seeks to assign a date to each node on the phylogeny representing the ‘best estimate’ dating for the species divergence represented by that node. Existing methods for phylogenetic dating are based on the idea of a ‘molecular clock’. Specifically, phylogenetic dating methods make use of available date estimates (e.g. from dated fossils), which provide time calibrations for one or more nodes of an input undated phylogeny, and combine these known calibrations with an underlying model for evolutionary rates variation along the branches of the phylogeny to estimate a date for every internal node of the phylogeny.

The first methods for phylogenetic dating were based on the idea of a ‘strict’ molecular clock, implying a uniform rate of evolution along different lineages (Langley and Fitch, 1974; Zuckerkandl and Pauling, 1962). However, researchers soon observed that different lineages in a species phylogeny can evolve at different rates and more complex methods based on ‘relaxed’ or ‘local’ molecular clock models were introduced to address this gap (Dos Reis *et al.*, 2016; Drummond *et al.*, 2006; Rambaut and Bromham, 1998; Renner *et al.*, 2008; Sanderson, 2002; Tamura *et al.*, 2012; Thorne *et al.*, 1998; Volz and Frost, 2017; Yang and Rannala, 2006; Yoder and Yang, 2000). Among all existing methods for phylogenetic dating, Bayesian approaches are generally believed to be the most accurate (Bouckaert *et al.*, 2014; Dos Reis *et al.*, 2016; Drummond *et al.*, 2006; Lartillot and Philippe, 2004; Yang and Rannala, 2006). Such Bayesian approaches are widely used due to their ability to implement more complex evolutionary models and account for inference uncertainty by sampling from the posterior. Recently, approaches based on an error-correction approach, rather than on an explicit relaxed molecular clock model, have also been proposed (Mai and Mirarab, 2020; To *et al.*, 2016). Such approaches aim to compute minimum deviations from the strict molecular clock model along tree edges to infer a chronogram satisfying given time calibrations.

**Fig. 1.**
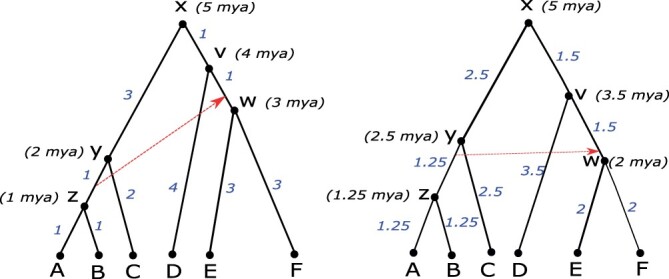
Chronograms and relative time calibrations. The figure shows two distinct chronograms for the same underlying phylogenetic tree. Each chronogram is dated backward in time, with the six leaves (tips) representing contemporary nodes. Dates assigned to internal nodes are shown italicized within parentheses and are in units of ‘mya’. Implied branch lengths are shown italicized in blue along edges. The dotted red line in each chronogram represents a known HGT event from the edge (*y*, *z*) to the edge (*v*, *w*). This horizontal transfer provides a relative time calibration, implying that node *y* must be older than node *w*. The chronogram on the left is not consistent with this relative time calibration while the chronogram on the right is

A key limitation of all of these phylogenetic dating methods is that they can only take *absolute* time calibrations into consideration. An absolute time calibration is one that constrains the date of a particular node of the phylogeny, generally based on the fossil record. Such constraints typically take the form of lower and/or upper bounds on the dating for one or more of the nodes in the phylogeny; for example, an absolute time calibration might be that a particular internal node *x* should be dated to between 100 and 120 million years ago (mya). Thus, these methods cannot take into account *relative* time calibrations that impose relative temporal orderings on two or more nodes of the phylogeny; for example, a relative time calibration might be that a node *x* must be dated to be older than a node *y* (where *x* is not an ancestor of *y*). However, using relative time calibrations is important when dating microbial phylogenies. This is primarily due to two reasons: First, microbial fossil data are extremely rare, making it difficult to use only absolute time calibrations to reliably date microbial phylogenies. Second, widespread horizontal gene transfer (HGT or transfer for short) between microbes makes it possible to obtain reliable relative time calibrations in many cases (e.g. Davín *et al.*, 2018; Gogarten *et al.*, 1999; Magnabosco *et al.*, 2018). Specifically, a transfer from edge (*a*, *b*) to edge (*c*, *d*) in the phylogeny, where *a* is the parent of *b* and *c* is the parent of *d*, can only occur if *a* is older than *d*. This is illustrated in Figure 1.

Related previous work. It has been observed that chronograms constructed using only absolute time calibrations often violate known (high-confidence/hand-curated) relative time calibrations. This has motivated the development of some recent approaches (Davín *et al.*, 2018; Fournier *et al.*, 2021; Gruen *et al.*, 2019; Magnabosco *et al.*, 2018; Wolfe and Fournier, 2018) for using transfer-based relative time calibrations to improve phylogenetic dating. Davín *et al.* (2018) explored the potential of using large numbers of computationally inferred transfer events to relatively order all nodes of a phylogeny by time and found that relative orderings computed this way were largely consistent with estimates from relaxed molecular clock models. Gruen *et al.* (2019) and Wolfe and Fournier (2018) demonstrated an alternative approach, directly calibrating gene trees in the presence of HGT, so that fossils from distantly related groups were able to place active constraints on HGT donor and recipient lineages. However, due to a dependence upon calibrations being available for groups directly involved in HGT, and the limited sequence information for dating within individual gene trees, this approach is likely only useful for dating a small subset of microbial groups. More recently, Fournier *et al.* (2021) developed a distinct relative dating approach, first applying standard Bayesian molecular clock analysis based on appropriate absolute calibrations, and then incorporating knowledge of relative calibrations by only sampling those chronograms from the posterior that satisfy all or most of the relative calibrations. However, with such a Bayesian rejection-sampling approach, it is possible that none of the sampled chronograms are compatible with all (or even most) of the relative calibrations. Therefore, this approach is only useful when the posterior space explored includes a robust sampling of HGT-consistent chronogram solutions, which may not be the case if some lineages are evolving at widely differing rates, unconstrained by local fossil calibrations. Finally, in recently published independent work, Szollosi *et al.* (2022) introduced a Bayesian approach that combines absolute and relative time calibrations into a single phylogenetic dating framework, allowing for the sampling of chronograms satisfying both absolute and relative time calibrations. While the work of Szollosi *et al.* (2022) has the same overarching goal as our proposed approach, the two approaches are methodologically distinct. We discuss the implications of these methodological differences in detail in Section 5.

Our contribution. We propose a novel approach, called DaTeR (short for Dating Trees using Relative constraints), for dating microbial species trees that address the limitations of existing approaches. In a sense, DaTeR is inspired by the Bayesian rejection-sampling idea of Fournier *et al.* (2021): instead of rejecting sampled chronograms that are incompatible with the given relative calibrations, DaTeR minimally ‘error-corrects’ each sample to ensure compatibility with all relative time calibrations. Specifically, we use a constrained optimization framework where we compute a minimal deviation from assigned node dates or branch lengths (representing time), under several appropriately designed objective functions, for the posterior chronogram sample such that all relative constraints are satisfied. We propose three different objective functions to appropriately measure deviation from an input chronogram: the first minimizes the weighted sum of squared differences between corresponding branch lengths in the input and error-corrected chronograms, the second minimizes the weighted sum of squared log ratios of the corresponding branch lengths and the third minimizes the weighted sum of squared differences between corresponding internal node dates. We evaluated our approach on a real biological dataset of 170 microbial species (mainly Cyanobacteria) and a reliable set of 24 transfer-based relative time calibrations used in a previous study (Fournier *et al.*, 2021), under six different molecular dating models. Among other findings, we observed that (i) chronograms constructed without considering relative time calibrations often violate several relative constraints, (ii) DaTeR is able to satisfy all relative constraints by making minor adjustments to the input chronograms, (iii) the different objective functions implemented in DaTeR can help fine-tune DaTeR to the characteristics of the dataset being analyzed, (iv) DaTeR can be used to help select the most appropriate molecular dating model to use to compute the most accurate initial posterior chronogram estimates, (v) DaTeR substantially improves upon the Bayesian rejection-sampling approach and (vi) DaTeR is highly scalable, requiring less than second on each input chronogram sample under two of the three objective functions. Overall, our results demonstrate that DaTeR is effective and scalable and that its application can have a significant impact on inferred dates.

The remainder of this article is organized as follows: basic definitions and preliminaries appear in Section 2, DaTeR is described in Section 3, experimental results are shown in Section 4 and concluding remarks appear in Section 5.

## 2 Definitions and preliminaries

A *chronogram* is a rooted binary tree in which each node of the tree has an assigned date. (Equivalently, a chronogram may be viewed as a rooted binary tree in which branch lengths represent time units. These branch lengths immediately imply a date for each internal node of the tree.) We assume that nodes are dated backward in time from the leaves (i.e. tips, representing terminal taxa) of the tree to the root. Such dates are often expressed in units of ‘mya’ and represent either known sampling times or estimated divergence times. In most cases, the leaves of the tree represent contemporary species and are assigned a date of 0. An illustration appears in Figure 1.

Given a chronogram *T* on *n* leaves, we label the internal nodes of *T*, starting from the root, as 〈1,2,…,n−1〉 and then label the leaves as 〈n,n+1,…,2n−1〉. The date of node *i*, where 1≤i≤2n−1, is then denoted by *t_i_*. We use *p*(*i*) to denote the parent node of *i* in *T*. We can also assign a branch length to each edge of *T* representing the time span between its two end points: For any i∈{2,…,2n−1} (i.e. for any non-root node *i*), we denote the branch length of edge (p(i),i) by *b_i_* where $b_{i}=|t_{p(i)}-t_{i}|$.

Constraints on chronograms. The following three types of constraints can be imposed on dates assigned to nodes in a chronogram.

1. Topological temporal constraints. These constraints require that the parent of a node *i* must be dated to be at least as old as the node *i* itself, that is, $t_{p(i)}\geq t_{i}$. Any valid chronogram must, at a minimum, satisfy all such constraints.
2. Absolute time constraints. This type of constraint is generally obtained from fossil data and imposes an upper and/or lower bound on the date assigned to a specific node in the chronogram. Such constraints may be available for one or more of the nodes in the phylogeny and, if imposed, the corresponding chronogram is required to satisfy all such constraints.
3. Relative time constraints. This type of constraint imposes a relative temporal ordering between two nodes not directly related by an ancestor–descendant relationship. For example, such a constraint may require that a node *i* be dated to be at least as old as another node *j*, even though *i* is not an ancestor of *j* in the phylogeny. Such constraints are generally based on known or inferred HGT events and several such constraints, each constraining the relative dating of two nodes, may be available for the phylogeny being dated. Figure 1 depicts an example of a relative constraint.

Dating using both absolute and relative time constraints. Recall that traditional methods for phylogenetic dating are unable to use relative time constraints and that recently proposed approaches for incorporating information from relative time constraints into phylogenetic dating have some fundamental limitations. Our new approach, DaTeR, addresses these limitations and allows for the use of both absolute and relative time constraints for improved phylogenetic dating.

DaTeR uses a two-step approach that first leverages existing methods for phylogenetic dating using absolute time constraints to construct an initial sample of chronograms and then minimally error-corrects those chronograms to make them compatible with all given relative time constraints. Such a two-step approach has the advantage that it can leverage any current or future framework for dating with absolute time constraints, allowing for the use of the most appropriate and/or most sophisticated dating models for constructing the initial chronogram estimates. The error-correction-based framework of DaTeR is inspired by recently developed approaches for phylogenetic dating that view the problem as one of error-correcting the branch lengths to make the inferred chronogram compatible with all given absolute time constraints (Mai and Mirarab, 2020; To *et al.*, 2016). In DaTeR, we apply error-correction in the context of relative time constraints rather than absolute time constraints.

## 3 Methodological details

### 3.1 Overview of DaTeR

DaTeR takes as input a collection of chronograms sampled from the posterior using any standard Bayesian phylogenetic dating approach under a suitable evolutionary model calibrated using suitable absolute time constraints. Thus, these sampled chronograms represent best estimates based on using only absolute time constraints. (We point out that an absolute constraint need not always take the form of a strict lower and/or upper bound and can instead take the form of some probability distribution on node dates. Any such existing model of time calibration using absolute constraints can be used when computing input chronogram samples.) In addition to these sampled chronograms, DaTeR also takes as input a set of relative time constraints. We use $T_{1},\ldots ,T_{m}$ to denote the *m* input chronograms and *R* to denote the set of relative time constraints. DaTeR then minimally error-corrects each input chronogram to make it compatible with all given relative temporal constraints. DaTeR implements three different *objective functions* to define and compute ‘minimally’ error-corrected chronograms: the first minimizes the sum of deviations of internal node dates between the input and error-corrected dating, the second minimizes the sum of squared deviations of branch lengths and the third minimizes the sum of squared log ratios of branch lengths. Once all input chronograms have been error-corrected under the chosen objective function, a single representative chronogram is computed by aggregating across all error-corrected chronograms.

Algorithm. DaTeR ($T_{1},\ldots ,T_{m},R$)

1: Verify that all relative constraints in *R* are temporally consistent.

2: Let O denote the objective function to be used.

3: **for** each k∈{1,…,m}**do**

4:  Let $T\prime_{k}$ be the chronogram obtained by minimally error-correcting *T_k_* with respect to objective function O such that $T\prime_{k}$ satisfies all relative constraints in *R*.

5: **end for**

6: Compute a final chronogram $T\prime_{\text{OPT}}$ by averaging dates across all error-corrected chronograms. Specifically, for a node *i* of $T\prime_{\text{OPT}}$, we assign the average of dates assigned to node *i* across all $T\prime_{1},\ldots T\prime_{m}$.

7: **return**$T\prime_{\text{OPT}}$

Observe that DaTeR first ensures (Step 1) that the relative constraints provided in *R* are compatible with each other. This can be verified efficiently by checking for the presence of a directed cycle (indicating temporal conflict) in the graph representation of the constraints (Tofigh *et al.*, 2011). Note that DaTeR can be used not only to compute a single aggregate dating $T\prime_{\text{OPT}}$, but also to obtain distributions of dates for each node based on the error-corrected chronograms $T\prime_{1},\ldots T\prime_{k}$.

Next, we describe the three objective functions implemented in DaTeR.

### 3.2 Objective functions

Given an input chronogram *T* and the set of relative constraints *R*, the proposed objective functions seek to compute a minimal deviation from chronogram *T* such that all relative constraints in *R* are satisfied, as described below.

Minimum squared branch length deviation (SBD). In the SBD objective function, the goal is to minimize the weighted sum of squared deviations of branch lengths between the input and output chronograms. Let *T* and T′ denote the input and output chronograms, respectively, on *n* leaves. Let *t_i_* represent the date assigned to node *i* in *T* and $t\prime_{i}$ denote date assigned to node *i* in T′, for each i∈{2,…,2n−1}. Then, the deviation of branch lengths for the edge (p(i),i) is given by $\left|(t_{p(i)}-t_{i})-(t\prime_{p(i)}-t\prime_{i})\right|$. The SBD objective function minimizes the weighted sum of SBDs across all edges of the input chronogram. We weight each term in this summation by the inverse of the input branch length $\frac{1}{t_{p(i)}-t_{i}}$. This weighting scheme helps minimize large deviations across short branches; that is, it helps ensure that the same magnitude of deviation can occur more easily along longer branches than shorter ones. This SBD objective function is similar to the objective function used in the LSD method (To *et al.*, 2016) in the context of absolute time constraints. Formally, the SBD objective function seeks to minimize the following:

(1) $$\phi (T,t\prime_{1},\ldots ,t\prime_{2n-1})=\sum_{i=2}^{2n-1}\frac{1}{t_{p(i)}-t_{i}}{\left(\left(t_{p(i)}-t_{i})-(t\prime_{p(i)}-t\prime_{i}\right)\right)}^{2}.$$

Minimum squared log ratio of branch lengths (SLRB). Instead of considering differences between branch lengths, as in SBD, the SLRB objective function considers the squared log of the ratio of the two branch lengths, that is, ${\;\text{log}\;}^{2}\frac{t\prime_{p(i)-t\prime_{i}}}{t_{p(i)}-t_{i}}$. Considering the ratio of branch lengths makes intuitive sense since it is robust to actual edge lengths and instead captures the change in evolutionary rate along an edge. Taking the square of the log of this ratio ensures that the same cost is applied irrespective of whether the new branch length is larger or smaller (multiplied or divided by the same scalar value). The SLRB objective function minimizes the weighted sum of squared log ratios across all edges of the input chronogram. This objective function is inspired by the LogDate method of Mai and Mirarab (2020) for dating with absolute time constraints and, like in LogDate, we weight each term in this summation by the square root of the input branch length $\sqrt{t_{p(i)}-t_{i}}$. Formally, the SLRB objective function seeks to minimize the following:

(2) $$\phi (T,t\prime_{1},\ldots ,t\prime_{2n-1})=\sum_{i=2}^{2n-1}\sqrt{t_{p(i)}-t_{i}}{\;\text{log}\;}^{2}\frac{t\prime_{p(i)}-t\prime_{i}}{t_{p(i)}-t_{i}}.$$

Minimum squared date deviation (SDD). The two previous objective functions consider changes in branch lengths. In contrast, the SDD objective function directly considers deviation in dates assigned to nodes in the input and output chronograms. The deviation in dates assigned to node *i* is given by $\left|(t_{i}-t\prime_{i})\right|$ and the SDD objective function minimizes the weighted sum of SDDs across all internal nodes of the input chronogram. We weight each term in this summation by the inverse of the date in the input chronogram $\frac{1}{t_{i}}$. This weighting scheme helps ensure that larger date deviations occur more easily along older nodes of the chronogram than along more recent nodes. Formally, the SDD objective function seeks to minimize the following:

(3) $$\phi (T,t\prime_{1},\ldots ,t\prime_{2n-1})=\sum_{i=1}^{n-1}\frac{1}{t_{i}}{\left(t_{i}-t\prime_{i}\right)}^{2}.$$

**Fig. 2.**
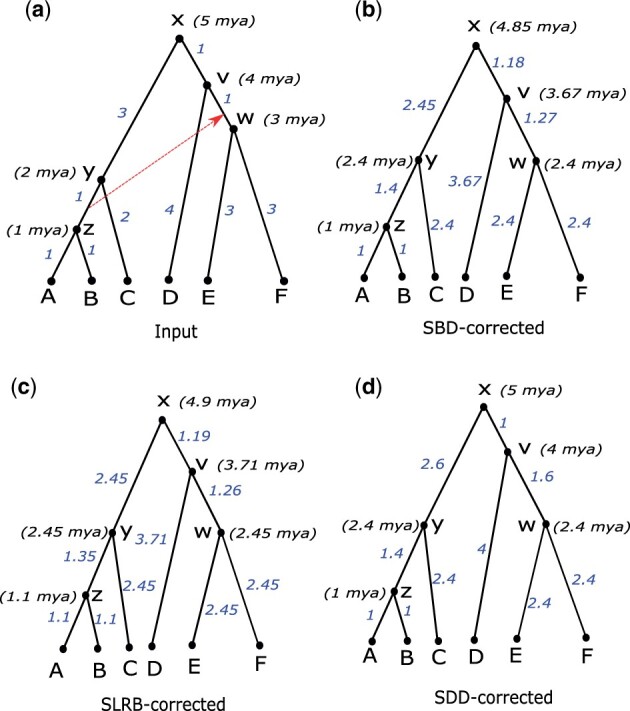
Illustration of objective functions implemented in DaTeR. (**a**) An input chronogram which is not consistent with the depicted horizontal transfer event (dotted red line) implying that *y* should be dated to be at least as old as *w*. The chronograms shown in parts (**b**), (**c**), and (**d**) are obtained by error-correcting the chronogram in (a) using DaTeR under SBD, SLRB and SDD objective functions, respectively. Observe that each error-corrected chronogram satisfies the relative constraint and that each of the three objective functions results in a slightly different chronogram

Figure 2 illustrates the application of these three objective functions and demonstrates how, given the same input chronogram, they can result in substantially different output chronograms. Figure 2 also illustrates how, when error-correcting a single input chronogram, DaTeR has a tendency to just barely satisfy each relative time constraint. Specifically, if a relative constraint that is initially violated in the input chronogram requires that node *i* be dated to be at least as old as node *j*, then the output, error-corrected chronogram may assign the same date to nodes *i* and *j*, so that the required constraint is just barely satisfied. However, as we demonstrate later, this tendency is suppressed when multiple chronograms are taken as input and results are aggregated across all the corresponding error-corrected chronograms. This is because different input chronogram samples vary in the specific rates and resulting time intervals assigned to branches. Consequently, different chronogram samples often violate different sets of constraints. Conversely, for any relative constraint, there are likely many input chronograms that already satisfy that constraint and will be included in the posterior sampling as unequal ages. This allows for the two nodes involved in such a constraint to have different date assignments in DaTeR’s final aggregated output. We point out, however, that if a relative time constraint is violated by all input chronograms, then the two nodes involved in that constraint may be assigned the same dates even in the aggregated output.

### 3.3 Formulation as a constrained optimization problem

DaTer uses a constrained optimization framework to compute error-corrected chronograms, that is, to implement Step 4 of Algorithm DaTeR, under the three objective functions described above.

Constrained optimization objective. Given a single input chronogram *T* and set *R* of relative time constraints, DaTeR seeks to find the arguments $\left\{t\prime_{1},\ldots ,t\prime_{2n-1}\right\}$ that minimize $\phi (T,t\prime_{1},\ldots ,t\prime_{2n-1})$ under the selected function (Equations 1, 2 or 3).

Constraints. There are three categories of constraints as listed below.

1. For each non-root node *i*, where 2≤i≤2n−1, we include the following topological temporal constraint: $t\prime_{p(i)}>t\prime_{i}$.
2. For each relative time constraint from *R* requiring that node *i* be dated at least as old as node *j*, we include the following constraint: $t\prime_{i}\geq t\prime_{j}$.
3. Finally, we assume, by default, that the dates of the leaf nodes in the chronogram are known and therefore fixed. This results in the following constraint for each leaf node *i*, where n≤i≤2n−1: $t\prime_{i}=t_{i}$.

Optional constraints. If desired, constraints can also be added corresponding to known absolute time constraints used when inferring the input chronogram *T*. By default, we do not include any absolute time constraints in the constrained optimization framework since the addition of such constraints can, in principle, make it impossible to also simultaneously satisfy all relative time constraints.

Computing optimal solutions. To solve the above non-linear programming formulations, DaTeR uses IBM ILOG CPLEX Optimization Studio through DOcplex API version 2.23 for Python to implement SBD and SDD. Since CPLEX does not support the log  operation as required by SLRB, DaTeR uses the ‘trust-constr’ method implemented in SciPy (Virtanen *et al.*, 2020) to implement SLRB. We note that the SBD and SDD problem formulations are expected to be convex, as with LSD (To *et al.*, 2016). However, as with LogDate (Mai and Mirarab, 2020), SLRB is expected to be non-convex.

### 3.4 Aggregating over all input chronograms

DaTeR takes as input *m* chronogram estimates and computes a final chronogram, $T\prime_{\text{OPT}}$, by aggregating across all *m* corresponding error-corrected chronograms (Step 6 of Algorithm DaTer). Specifically, for a node *i* of $T\prime_{\text{OPT}}$, we assign the average of dates assigned to node *i* across all *m* error-corrected chronograms $T\prime_{1},\ldots T\prime_{m}$. A similar aggregation is often performed when aggregating across multiple chronogram samples from the posterior of a Bayesian analysis and it is easy to see that the result of aggregating multiple chronograms of the same underlying phylogenetic tree must itself be a valid chronogram. Importantly, it can also be shown that if the error-corrected chronograms $T\prime_{1},\ldots T\prime_{m}$ are each consistent with the given relative time constraints, then so must the aggregated chronogram $T\prime_{\text{OPT}}$. We formally state this claim below. Its proof appears in the Supplement.

Lemma 3.1. *If* $T\prime_{1},\ldots T\prime_{m}$*each satisfy all relative constraints in R then* $T\prime_{\text{OPT}}$*satisfies all relative constraints in R.*

We point out that this result holds true for any summarization function (not just for *average*, as in the lemma above) which has the following monotonicity property: If two lists, *A* and *B*, of numbers have the same length and each entry of list *A* is no less than the corresponding entry in list *B*, then the summarization function applied to *A* yields a value that is no less than the value obtained by applying the same summarization function to *B*. Examples of such functions include *average*, *median*, *min*, *max*, etc.

## 4 Results

### 4.1 Dataset description

We applied DaTeR to a large, recently published dataset consisting of 170 Cyanobacterial genomes (Fournier *et al.*, 2021). This set of 170 Cyanobacteria genomes were manually curated for best representation of extant cyanobacterial diversity and a species tree topology was determined from a maximum-likelihood tree made using RAxML from a concatenated alignment of 30 ribosomal proteins, which was then used in our molecular clock analysis (Fournier *et al.*, 2021). This dataset consists of 1000 posterior chronogram samples for the species tree, computed using PhyloBayes (Lartillot and Philippe, 2004) under each of six different molecular dating models (for a total of 6000 chronogram samples). The six molecular dating models used are: (i) uncorrelated gamma multipliers model (Drummond *et al.*, 2006) with birth–death priors (UGAM_bd), (ii) uncorrelated gamma multipliers model with uniform tree priors (UGAM_nobd), (iii) lognormal autocorrelated model (Thorne *et al.*, 1998) with birth–death priors (LN_bd), (iv) lognormal autocorrelated model with uniform tree priors (LN_nobd), (v) Cox–Ingersoll–Ross process model (Lepage *et al.*, 2007) with birth–death priors (CIR_bd) and (vi) Cox–Ingersoll–Ross process model with uniform tree priors (CIR_nobd). Further details on the construction of these initial 6000 chronograms appear in Supplementary Section S1.

The dataset also includes 24 relative time constraints for the Cyanobacterial species tree. These relative time constraints were obtained from a set of 24 manually curated HGTs (Fournier *et al.*, 2021). Further details on these relative constraints appear in Supplementary Section S1.

Basic statistics. None of the chronograms sampled under CIR_bd, LN_nobd, LN_bd, UGAM_nobd, and UGAM_bd, and only 5 of the 1000 trees sampled under CIR-nobd, are compatible with all 24 relative constraints. Table 1 shows the average number of relative constraints violated by the 1000 sampled chronograms under each of the six different molecular dating models, along with the numbers of samples satisfying at least 23 of the 24 constraints. As the table shows, chronograms estimated under the CIR-nobd model are, on average, the most compatible with the 24 relative constraints, with 122 of the 1000 samples compatible with at least 23 of the 24 relative constraints. In contrast, chronograms estimated under the LN-bd model are the least compatible, violating, on average 9.14 relative constraints and with none of the 1000 chronograms compatible with at least 23 of the relative constraints. All models dated the root node to between 3885.80 and 3900.85 mya, on average.

**Table 1.** Basic statistics for input chronograms

Model	Average number of relative constraints violated	Number of samples satisfying ≥23 relative constraints
CIR_nobd	3.45	122
CIR_bd	5.808	6
LN_nobd	6.74	0
LN_bd	9.14	0
UGAM_nobd	7.25	0
UGAM_bd	6.78	0

*Notes*: The table shows two different measures of compatibility for the 1000 chronograms sampled under each of the six molecular dating models with the 24 relative constraints.

Error-correction using DaTeR. We used all 24 relative time constraints and applied DaTeR to the 1000 chronogram samples from each of the six molecular dating models. For each molecular dating model, we used each of the three objective functions implemented in DaTeR, so that 18 000 chronograms were error-corrected in total. We refer to these chronograms as DaTeR-corrected chronograms. Thus, after aggregation across the 1000 DaTeR-corrected chronograms for each dating model and objective function, we obtained 18 final DaTeR-corrected chronograms (three for each of the six molecular dating models).

### 4.2 Impact of DaTeR on inferred node ages

**Fig. 3.**
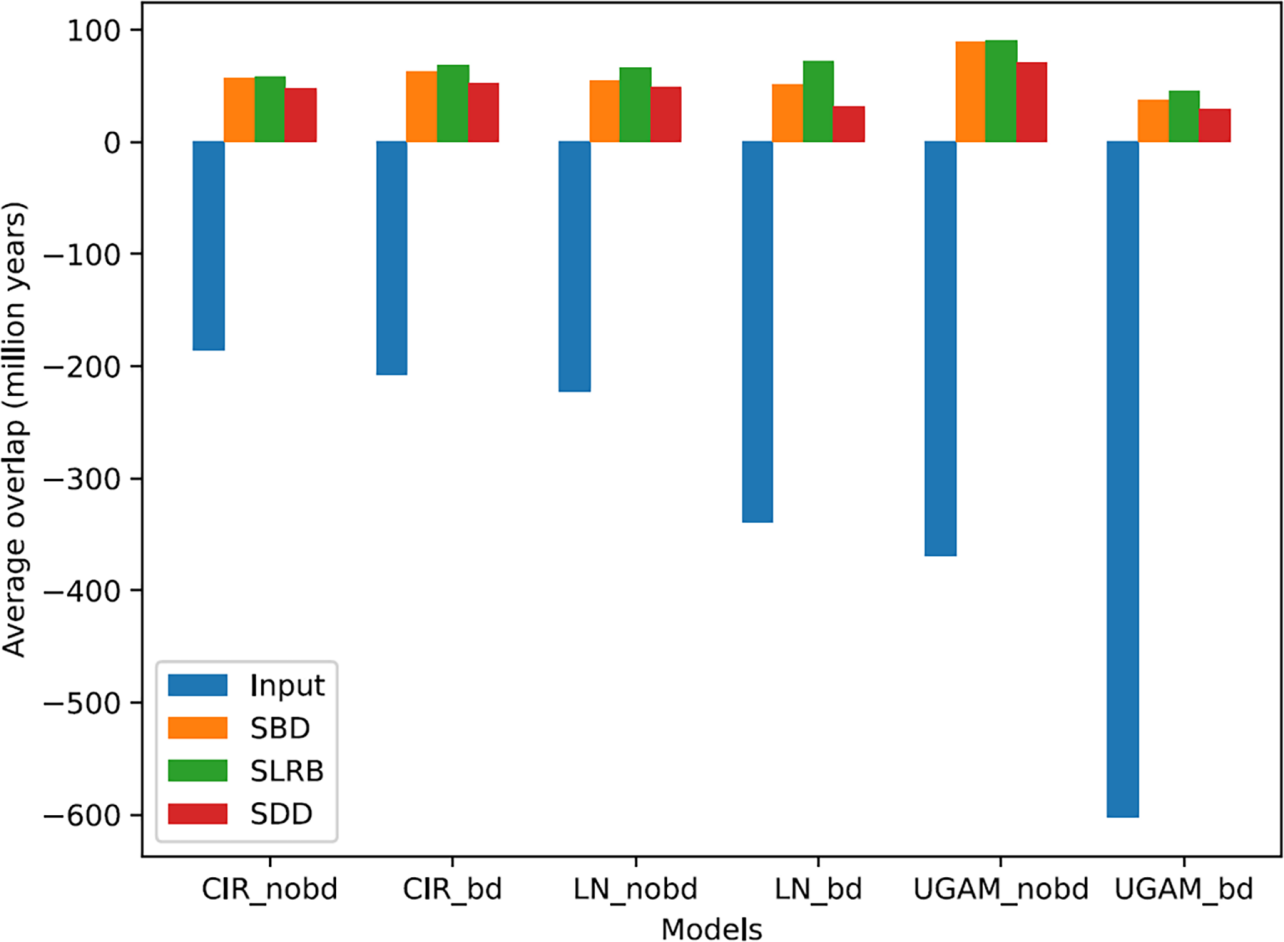
Average date gap for violated relative constraints. The figure shows the average difference in assigned dates for pairs of nodes corresponding to violated relative constraints in the aggregated input chronograms. A negative difference mean that the constraint is violated (i.e. that the difference in dates is in the wrong direction). Positive differences mean that the constraints are satisfied and the differences in assigned dates are in the correct direction. Results are shown for the aggregated input chronograms and the aggregated chronograms computed using the three objective functions implemented in DaTeR, for each of the six dating models

To assess the impact of DaTeR on chronogram estimation, we compared the DaTeR-corrected chronograms against the uncorrected input chronograms. As expected, each of the 18 000 DaTeR-corrected chronograms was compatible with all 24 relative constraints. To assess the change in node dates pre- and post-error-correction, we compared the six aggregated input chronograms against the 18 aggregated DaTeR-corrected chronograms. Figure 3 shows the average gap (difference in dates) between pairs of nodes corresponding to violated relative constraints in the aggregated input chronograms. As the figure shows, the substantial negative gap for the input chronograms, under all six models, is corrected and reversed by DaTeR under each molecular dating model and each objective function. The average correction for these node pairs is slightly under 250 million years under the CIR_nobd model and over 600 million years under the UGAM_bd model.

We also assessed the overall change in node dates and branch lengths between the input and DaTeR-corrected chronograms. Table 2 shows the results of this analysis. As the table shows, the average change in assigned node dates and branch lengths remains low across all three objective functions under the CIR_nobd model, but is substantially higher under the UGAM_bd and LN_bd models. Overall, these results demonstrate that, under appropriate models of molecular dating such as CIR_nobd, DaTeR can achieve compatibility with all relative constraints with only minor adjustments to node dates and branch lengths.

**Table 2.** Average differences in node dates and branch lengths

Model	Node date deviation			Branch length deviation		
	SBD	SLRB	SDD	SBD	SLRB	SDD
CIR_nobd	18.67	19.37	**5.09**	12.99	13.43	**6.48**
CIR_bd	31.1	29.81	**11.05**	21.2	20.48	**12.83**
LN_nobd	37.52	39.44	**13.06**	25.73	26.57	**14.98**
LN_bd	65.27	70.32	**24.15**	40.44	42.29	**25.57**
UGAM_nobd	59.85	58.49	**23.79**	40.0	39.02	**26.28**
UGAM_bd	67.36	58.08	**28.12**	42.93	39.58	**29.01**

*Notes*: Average differences between assigned node dates and branch lengths are shown between aggregated input chronograms and corresponding DaTeR-corrected chronograms. Results are shown for all three objective functions implemented in DaTeR and for all six molecular dating models. Node date deviation results are averaged over the 169 internal nodes in each chronogram and branch length deviation results are averaged over all 338 edges in each chronogram. Bold text indicates lowest (best) values.

### 4.3 Comparison across objective functions

**Fig. 4.**
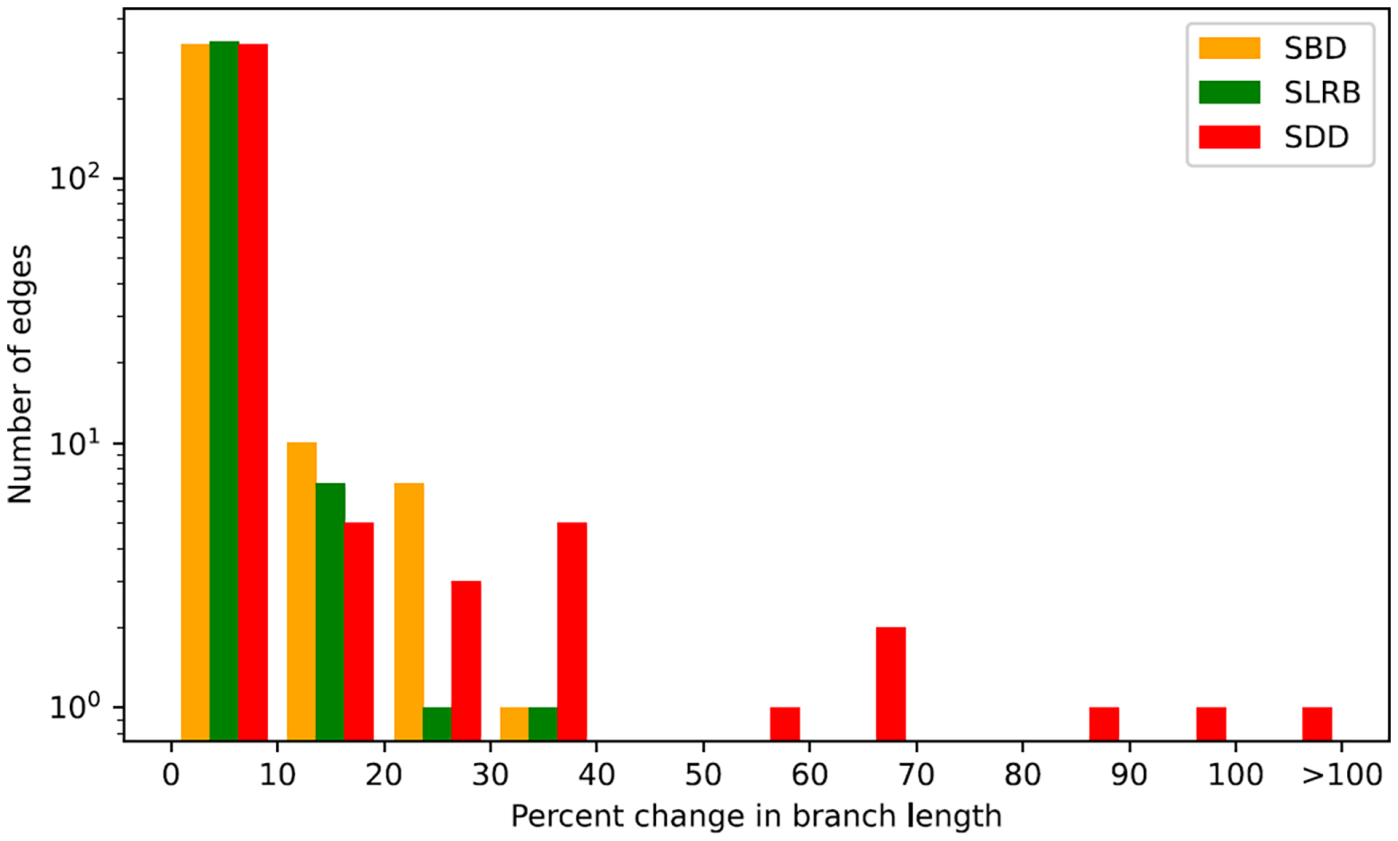
Bar chart of percent change in branch lengths. The number of edges within various percent ranges is shown for aggregated DaTeR-corrected chronograms under the three different objective functions for the CIR_nobd model. All intervals are left-closed and right-open, and a log scale is used for the *Y*-axis. The total number of edges is 338

As Table 2 shows, the three objective functions implemented in DaTeR result in different chronogram estimates. In particular, DaTeR with the SDD objective function results in significantly smaller deviations for both node dates and branch lengths compared with the SBD and SLRB objective functions. However, this does not necessarily imply that the SDD objective function results in more accurate chronograms. Specifically, an error-corrected chronogram in which input branch lengths are only minimally rescaled may be preferable to an error-corrected chronogram with lower overall node date or branch length deviation but greater branch-length rescaling. For instance, in many cases, it may be preferable to rescale a length 10 edge to length 12 (corresponding to a 20% change) instead of rescaling a length 2 edge to length 3 (corresponding to a 50% change). We therefore computed the average percent change in branch lengths for DaTeR-corrected chronograms under the three different objective functions and the left half of Table 3 shows these results. We find that the percent change is smallest for SLRB under all models and is largest for SDD under four of the six dating models. For example, under the CIR_nobd model, SLRB shows an average change of 1.69% while SBD and SDD show percent changes of 2.17% and 2.62%, respectively. A more fine-grained analysis appears in Figure 4, which shows how, for CIR_nobd, the majority of edges are rescaled only minimally, how SBD and SLRB do not rescale any edge by more than 40% (and SLRB rescales only 2 edges more than 20%) and how using SDD results in far more extreme rescaling of branch lengths for multiple edges. Thus, SLRB performs best under this measure despite resulting in larger overall changes in node dates and branch lengths.

**Table 3.** Percent change in branch lengths and pairwise node date deviations for different objective functions

Model	SBD (%)	SLRB (%)	SDD (%)	SBD versus SLRB	SDD versus SBD	SDD versus SLRB
CIR_nobd	2.17	**1.69**	2.62	**7.77**	13.13	13.94
CIR_bd	4.18	**3.0**	4.73	**14.63**	20.42	22.03
LN_nobd	3.99	**3.11**	4.95	**19.6**	24.56	28.92
LN_bd	7.34	**6.15**	8.85	**28.34**	41.22	53.17
UGAM_nobd	6.68	**5.5**	5.82	**32.89**	37.33	43.35
UGAM_bd	8.16	**7.28**	7.34	**37.83**	40.65	41.67

*Notes:* The left half shows the average percent change in branch lengths for aggregated DaTeR-corrected chronograms under the three different objective functions. The percent change in the branch length for an edge (p(i),i) is calculated as $\frac{\left|(t\prime_{p(i)}-t\prime_{i})-(t_{p(i)}-t_{i})\right|}{t_{p(i)}-t_{i}}\times 100$, where *t_j_* and $t\prime_{j}$ refer to the dates assigned to node *j* in the aggregated input chronogram and corresponding DaTeR-corrected chronogram, respectively. The right half shows average differences between assigned node dates between pairs of DaTeR-corrected aggregated chronograms computed using different objective functions. Results are shown for all six molecular dating models. Bold text indicates lowest (best) values.

We also assessed the overall pairwise similarities of the aggregated chronograms resulting from DaTeR-correction under the three objectives. As the right half of Table 3 shows, chronograms constructed using SBD and SLRB show significantly greater pairwise similarity to each other than they show to chronograms constructed using SDD.

Further assessment using simulated data. To gain further insight into the behavior of these objective functions, we applied DaTeR to simulated datasets with known node dates. We used the phylogenetic simulation software SaGePhy (Kundu and Bansal, 2019) to first generate 20 simulated ultrametric species trees, each with 100 leaves and height 1 and evolved under a birth–death model, and then relaxed the branches of these species trees using autocorrelated lognormal rate scaling under the model of Rannala and Yang (2007). For 10 of the 20 species trees, we performed this scaling using a start rate of 1 and $\sigma^{2}=0.25$, and for the remaining 10 species trees we used a start rate of 1 and $\sigma^{2}=0.5$. This resulted in two sets of scaled species trees, each consisting of 10 trees.

We then used the phylogenetic dating method LogDate (Mai and Mirarab, 2020) with default parameters, together with a single absolute time constraint specifying the age of the root node to be 1, to infer a single chronogram from each of the 20 scaled species trees. These chronograms were then error-corrected using additional relative time constraints under each of the three objective functions implemented in DaTeR, and the assigned node dates were compared with the node dates in the corresponding original ultrametric species tree. We used randomly generated HGTs to obtain the relative time constraints used with each input chronogram when error-corrected using DaTeR. Specifically, we used SaGePhy (Kundu and Bansal, 2019) to generate a ‘gene’ tree with randomly invoked HGTs for each of the 20 original ultrametric species trees. This resulted in several relative time constraints per species tree, some of which were violated in the corresponding LogDate chronogram. We used these violated relative time constraints when error-correcting the LogDate chronograms with DaTeR, ensuring that there were at least three violated relative time constraints for each LogDate chronogram. On average, we used 3.85 relative time constraints to error-correct each LogDate chronogram. The specific SaGePhy commands used to generate the initial ultrametric species trees, rescale their branch lengths and generate gene trees with HGTs are given in Supplementary Section S1.

We compared the accuracies of assigned node dates in the error-corrected chronograms inferred using the three objective functions. On the 10 species trees scaled using parameters [1,0.25], the scaled trees, LogDate chronograms, SDD chronograms, SBD chronograms and SLRB chronograms showed total node date deviations of 7.8, 5.2, 4.84, 4.45 and 4.4, respectively, on average per tree. On the 10 species trees scaled using parameters [1,0.5], the scaled trees, LogDate chronograms, SDD chronograms, SBD chronograms and SLRB chronograms showed total node date deviations of 15.25, 7.67, 7.19, 6.475 and 6.52, respectively, on average per tree. These results show that while all three objective functions are able to improve upon the LogDate tree, these improvements are, as expected, greatest for the SBD and SLRB objective functions.

Overall, the above experimental results using both real and simulated data suggest that (i) SBD and SLRB may be preferable to SDD in many cases and that SLRB is a good ‘default’ objective function for DaTeR, (ii) the three objective functions result in non-identical chronogram estimates but SBD and SLRB generally result in similar chronograms and (iii) SDD can result in more distinct chronograms and may be preferable under certain scenarios, for example, when the goal is to minimize overall change in node dates or branch lengths.

### 4.4 Application to model selection

A key consideration in phylogenetic dating is the choice of molecular dating model to use. As can be seen above (Tables 1 and 2 and Fig. 3), the choice of molecular dating model directly impacts chronogram estimation. However, it can often be difficult to identify the most appropriate molecular dating model to use with a given dataset. Checking for compatibility with known relative constraints and assessing the magnitude of node date or branch length deviation required by DaTeR on chronograms estimated under different molecular dating models can help address this difficulty.

Indeed, using the results shown in Tables 2 and 3, we can easily identify CIR_nobd as the most appropriate molecular dating model for our dataset. Importantly, the superiority of CIR_nobd for this dataset is consistently identified under all three of DaTeR’s objective functions and all reported measures of deviation (node deviation, branch length deviation or percent change in branch lengths). All objective functions and measures of deviation also clearly identify UGAM_bd and LN_bd as the least appropriate models to use for this dataset. These insights are also compatible with the statistics on average numbers of relative constraints violated under each molecular dating model, where we find that CIR_nobd violates the fewest relative constraints and LN_bd violates the most. Thus, in addition to chronogram estimation under a given molecular dating model, DaTeR can be used to help select the most appropriate molecular dating model to use among the candidates under consideration.

### 4.5 Comparison against rejection-sampling approach

Fournier *et al.* (2021) previously analyzed this Cyanobacterial dataset to assess the impact of using relative time constraints on phylogenetic dating. They employed a Bayesian rejection-sampling approach where posterior chronogram chronograms samples (1000 chronograms sampled from the posterior using PhyloBayes, under six different molecular dating models) that did not satisfy at least a certain fraction of the 24 relative constraints were rejected (Fournier *et al.*, 2021). Recall that only 5 of the total of 6000 input chronogram samples, across all six models, are compatible with all 24 relative constraints (see also Table 1). Thus, Fournier *et al.* (2021) used lower relative constraint compatibility cutoffs in their analysis, balancing the need for sufficiently many chronogram samples with other considerations for the different models. For our comparative analysis, we applied Bayesian rejection sampling by rejecting those samples that satisfied fewer than 21 relative constraints. This resulted in a subset of chronogram samples that was large enough to reasonably estimate age distributions under most of the molecular dating models: 709 samples for CIR_nobd, 203 for CIR_bd, 57 for LN_nobd, 8 for LN_bd, 13 for UGAM_nobd and 8 for UGAM_bd.

We compared the final aggregated chronograms computed using DaTeR with those computed using this Bayesian rejection-sampling approach. As Table 4 shows, all Bayesian rejection-sampling aggregated chronograms violate between two and four relative constraints, with the chronogram computed under CIR_nobd violating the fewest. As the table shows, the constraints violated in these aggregated chronograms also show large average date gaps (in the sense of Fig. 3), with an average date gap of −224.01 under CIR_nobd. As with DaTeR (Table 2), we also computed average differences between assigned node dates and branch lengths between aggregated input chronograms and corresponding Bayesian rejection-sampling chronograms. As Table 4 shows, Bayesian rejection-sampling chronograms show significantly higher node date deviations and branch length deviations than DaTeR (under all objective functions) for five out of the six molecular dating models. This highlights DaTeR’s ability to satisfy more (all) relative constraints with less node date and branch length deviation.

**Table 4.** Results of applying Bayesian rejection sampling

Model	Rejection-sampling chronograms			
	Relative constraints violated	Average date gap	Node date deviation	Branch length deviation
CIR_nobd	2	−224.01	7.23	5.25
CIR_bd	3	−243.77	34.77	21.82
LN_nobd	3	−238.85	77.05	47.2
LN_bd	4	−134.17	154.75	93.02
UGAM_nobd	4	−343.82	70.11	53.18
UGAM_bd	3	−915.23	69.24	47.29

*Notes*: Basic summary statistics are shown for aggregated chronograms computed using the Bayesian rejection-sampling approach with a relative constraint cutoff of ≥21. The table shows the number of violated relative constraints, average difference in assigned dates for pairs of nodes corresponding to the violated relative constraints (as in Fig. 3) and average differences between assigned node dates and branch lengths between aggregated input chronograms and corresponding aggregated Bayesian rejection-sampling chronograms (compare with Table 2).

We also compared how assigned node dates differ between aggregated DaTeR-corrected chronograms, using the SLRB objective function, and corresponding Bayesian rejection-sampling chronograms. Supplementary Figures S1–S6 show the results for all six molecular dating models. We find that DaTeR and Bayesian rejection sampling show significant differences in assigned node dates for several nodes under all molecular dating models except CIR_nobd. Under the CIR_nobd model (Supplementary Fig. S1), both DaTeR and Bayesian rejection sampling assign similar dates for the majority of nodes (generally identical to input node dates), but DaTeR shows substantially higher deviation from input dates for a small subset of the nodes.

Finally, we compared the distribution of dates for each node reported by the two methods for each of the six molecular dating models. Recall that the input chronograms, DaTeR-corrected chronograms and Bayesian rejection-sampling chronograms all yield associated distributions of dates for each node (in addition to the mean/aggregated date for each node used in our analysis thus far). Supplementary Figures S7–S12 plot standard deviations for node dates for the six molecular dating models. As the plots show standard deviations for DaTeR-corrected chronograms are similar overall to those for the input chronograms but can vary from node to node, showing higher deviation compared with the input for some nodes and identical or lower for others.

### 4.6 Running time and scalability

DaTeR is fast and scalable and has minimal computational requirements. Supplementary Table S1 shows average running times of DaTeR per chronogram (averaged over the 1000 input chronogram samples) on a commodity desktop computer (Intel Core i5 3.2 GHz processor and 8 GB main memory) for each of the six molecular dating models. As the table shows, each run of DaTeR using the SBD or SDD objective function required less than a second per chronogram on our dataset (i.e. on chronograms with 170 leaves) for all six dating models. However, running times are significantly longer for SLRB, requiring an average of 33 min for each CIR_nobd chronogram and up to almost 2 h for each UGAM_bd chronogram. This drastic discrepancy in running time for SLRB versus the other two objective functions is likely due to the fact that DaTeR is able to use the highly optimized IBM ILOG CPLEX Optimization Studio to implement the SBD and SDD objective functions but uses the less optimized ‘trust-constr’ method implemented in SciPy (Virtanen *et al.*, 2020) to implement the SLRB objective function.

These results demonstrate how DaTeR, using SBD or SDD, can be used to error-correct and aggregate over hundreds or thousands of large chronograms in a matter of minutes. They also show how DaTeR can be feasibly applied to large chronograms, error-correcting and aggregating over hundreds of chronogram samples, within a few days even under the SLRB objective function. In fact, if multiple cores are available then this running time can be proportionally reduced through trivial parallelization (error-correcting different chronogram samples independently in parallel). Furthermore, if SLRB requires excessive running time, our previous results show that the similar and highly scalable SBD objective function can be used instead (Table 3).

## 5 Discussion and conclusion

In this work, we introduced a novel, error-correction-based approach, DaTeR, for estimating chronograms based on both absolute and relative time calibrations. Our extensive experimental analysis using a large empirical dataset, supplemented by a simulation study, demonstrates the potential of DaTeR for improved phylogenetic dating. We found that chronograms constructed using conventional approaches often violate known relative constraints and that DaTeR is able to satisfy all relative constraints by making minor adjustments to the input chronograms. We also found that DaTeR can be used to help select the most appropriate molecular dating model for computing initial posterior chronogram estimates and that DaTeR substantially improves upon the previously developed Bayesian rejection-sampling approach. Importantly, DaTeR is not only effective but also scalable, requiring less than a second on each input chronogram sample under two of the three objective functions. By allowing for the easy incorporation of relative time constraints, DaTeR will enable more accurate chronogram construction for microbial species.

DaTeR offers several advantages compared with the recently developed Bayesian approach of Szollosi *et al.* (2022). For example, a frequent challenge with Bayesian tree space exploration is that this space can be very small, or even non-existent, due to incompatibility with rate models or fossil calibrations or both. The method of Szollosi *et al.* (2022) cannot be used if one or more relative constraints are incompatible with the given node age calibrations, but DaTeR can rein in branch length behavior impacted by spurious or dubious by fossil calibrations and improve age estimates even in cases of incompatibility with fossil calibrations or rate models. Importantly, DaTeR can also be used with any molecular dating software and any dating model, approach or existing chronogram dataset, as well as dating models and approaches that have not yet been developed. In contrast, the method of Szollosi *et al.* (2022) relies upon a single implementation and is limited by the models and functionality available in RevBayes. Another key advantage of DaTeR is that it can be used to determine the best molecular clock branch rate model. The extent of error-correction needed to satisfy relative constraints can be used as a metric to assess the fitness of different molecular clock rate models. This novel *post hoc* assessment of molecular clock models helps address model fit and biases and can independently determine the most accurate model to use for retrieval of date estimates, which is a key issue in employing molecular clock models. Finally, DaTeR makes it quick and easy to assess the impact of adding or removing different relative constraints since the Bayesian molecular clock analysis does not need to be rerun. This allows for flexibility in the analysis as well as greater scalability.

DaTeR could be further improved in several different ways. Under the current formulation, when applying DaTeR to a single chronogram sample, the two nodes involved in a violated relative constraint are often assigned identical dates (i.e. a zero or almost zero date gap) in the resulting error-corrected chronogram. Thus, while DaTeR can also be used to error-correct single chronograms, such as those computed using fast phylogenetic dating methods like LSD (To *et al.*, 2016) or LogDate (Mai and Mirarab, 2020), the resulting error-corrected chronogram would exhibit a strong bias toward having fewer unique node date assignments (i.e. divergence times). Even when applying DaTeR to a collection of input chronograms and aggregating over the results, the resulting aggregated chronogram is biased toward having several pairs of nodes with similar node date assignments. Thus, chronograms estimated using DaTeR, though more accurate, show a particular bias in assigned dates. It would be useful to address this bias and enable larger date gaps between nodes corresponding to HGT-derived relative constraints. In addition, it may be beneficial to extend the framework to allow for a small number of relative constraints to be violated if satisfying such constraints requires large deviations from input dates or extreme rescaling of branch lengths. This could help account for any uncertainty or error in the utilized relative constraints. One approach to implementing the above improvements is to use ‘soft bounds’ for relative constraints. Such soft bounds methods exist for traditional phylogenetic dating and they allow for age estimates to slightly violate constraints based on a probability model (Bouckaert *et al.*, 2014; Yang and Rannala, 2006). This allows such methods to account for uncertainty/error in fossil calibrations and a similar idea could be applied to relative constraints. Finally, DaTeR’s underlying framework could be extended to simultaneously error-correct with respect to all given input chronogram samples and compute a single, globally optimal error-corrected chronogram, instead of aggregating across multiple error-corrected chronograms as in the current approach.

## Supplementary Material

btad084_Supplementary_Data

## Data Availability

The Cyanobacterial dataset analyzed in this work is publicly available (Fournier *et al.*, 2021). The specific commands used to generate simulated data are available in the Supplement. DaTeR software is freely available from https://compbio.engr.uconn.edu/software/dater/.
