# Supplementary material for: DaTeR: error-correcting phylogenetic chronograms using relative time constraints
Source: Bioinformatics. 2023 Feb 8;39(2):btad084. doi: 10.1093/bioinformatics/btad084 (PMC10075190; doi:10.1093/bioinformatics/btad084)
Supplement: btad084_Supplementary_Data [file btad084_supplementary_data.pdf]

Supplementary Material

S1 Supplementary Text

Proof of Lemma 3.1.

Proof. Consider any relative constraint of the form $t_i \geq t_j$ from R that is satisfied by all error-corrected chronograms $T'_1, \ldots T'_m$. It suffices to show that T'_{OPT} satisfies this relative constraint. For any node a, let t_a^k denote the date assigned to node a in chronogram T'_k, for $1 \leq k \leq m$. Then, $t_i^k \geq t_j^k$ holds true for each $k \in \{1, \ldots, m\}$. Summing up these inequalities we get

$$\sum_{k=1}^{m} t_i^k \geq \sum_{k=1}^{m} t_j^k,$$

which yields

$$\frac{\sum_{k=1}^{m} t_i^k}{m} \geq \frac{\sum_{k=1}^{m} t_j^k}{m}.$$

Observe that the left hand side of the above inequality is the date assigned to node i in the final aggregated chronogram T'_{OPT}, while the right hand side is the date assigned to node j. Thus, T'_{OPT} satisfies all relative constraints in R. □

Construction of initial chronograms. For our experimental analysis, we used, as input to DaTeR, the collection of 6000 uncalibrated chronograms constructed and used in Fournier et al. (2021). In that work, PhyloBayes 4.1c (Lartillot and Philippe, 2004) was used to perform molecular clock dating and for computing posterior date estimates, distributions, and chronograms. The analysis used C20 substitution models and the following six molecular dating models: (i) uncorrelated gamma multipliers model (Drummond et al., 2006) with birth-death priors (UGAM_bd), (ii) uncorrelated gamma multipliers model with uniform tree priors (UGAM_nobd), (iii) lognormal autocorrelated model (Thorne et al., 1998) with birth-death priors (LN_bd), (iv) lognormal autocorrelated model with uniform tree priors (LN_nobd), (v) Cox–Ingersoll–Ross process model (Lepage et al., 2007) with birth-death priors (CIR_bd), and (vi) Cox–Ingersoll–Ross process model with uniform tree priors (CIR_nobd). Two chains were run for each model, using the default automatic stopping rule. For burn in, 20% of chains were excluded before sampling 1,000 of the posterior trees. The molecular clock was calibrated using a root prior on the bacterial ancestor, with the date being normally distributed with a mean age of 3,900 mya (standard deviation of 200 mya). Additional fossil calibrations were not used for this analysis in order to observe the model effect of evolutionary rates across each chronogram, independent of node-specific constraints.

Estimation of relative constraints. We used the relative time constraints estimated by Fournier et al. (2021) for the Cyanobacterial dataset. These relative time constraints were estimated using high-quality, manually curated HGTs (Fournier et al., 2021). Specifically, index HGT candidates were filtered and identified using a distance based metric derived from the eggNOG 5.0 database of

clusters of orthologous groups (Huerta-Cepas et al., 2018), with subsequent phylogenetic reconciliation analysis and detailed reconstruction by manual curation, of candidate gene family clusters. IQtree (Nguyen et al., 2014) was used to run for best model fit trees, including bootstrap supports for confirmation. 24 unique pairs of donor and recipient internal nodes, representing clades, were then identified and used to obtain relative constraints between various phyla groups on the species tree. A full list of identified HGTs and corresponding relative time constraints appears in Fournier et al. (2021).

Commands used to generate simulated datasets.
SaGePhy (Kundu and Bansal, 2019) command used to generate each initial ultrametric species tree:

```
java -jar sagephy-1.0.0.jar HostTreeGen -min 100 -max 100 1.00 5.00 0.05 species
```

SaGePhy command used to rescale species tree branch lengths under the autocorrelated lognormal rate scaling model of Rannala and Yang (Rannala and Yang, 2007):

For parameters $[1, 0.25]$: `java -jar sagephy-1.0.0.jar BranchRelaxer -innms species.pruned.tree ACRY07 1 0.25 -o outputSpecies.txt`

For parameters $[1, 0.5]$: `java -jar sagephy-1.0.0.jar BranchRelaxer -innms species.pruned.tree ACRY07 1 0.5 -o outputSpecies.txt`

SaGePhy command used to generate gene trees with randomly invoked HGTs on each original ultrametric species tree:

```
java -jar sagephy-1.0.0.jar GuestTreeGen -db none -max 10000 -min 250 species.pruned.tree 0.6 1.8 1.2 geneTree
```

S2 Supplementary Figures and Tables

Model	Running time in seconds		
	SBD	SLRB	SDD
CIR_nobd	0.64	1981.40	0.75
CIR_bd	0.64	4728.25	0.76
LN_nobd	0.64	1747.87	0.74
LN_bd	0.64	2958.28	0.74
UGAM_nobd	0.64	3476.80	0.71
UGAM_bd	0.64	6792.55	0.71

Table S1: DaTeR running times. Average running times per chronogram are shown for DaTeR under all three objective functions. Results are shown for all six molecular dating models and are averaged over all 1000 input chronograms for each model. These timed runs were executed using a single core on a desktop computer with an Intel Core i5 3.2 GHz quad core processor and 8 GB main memory.

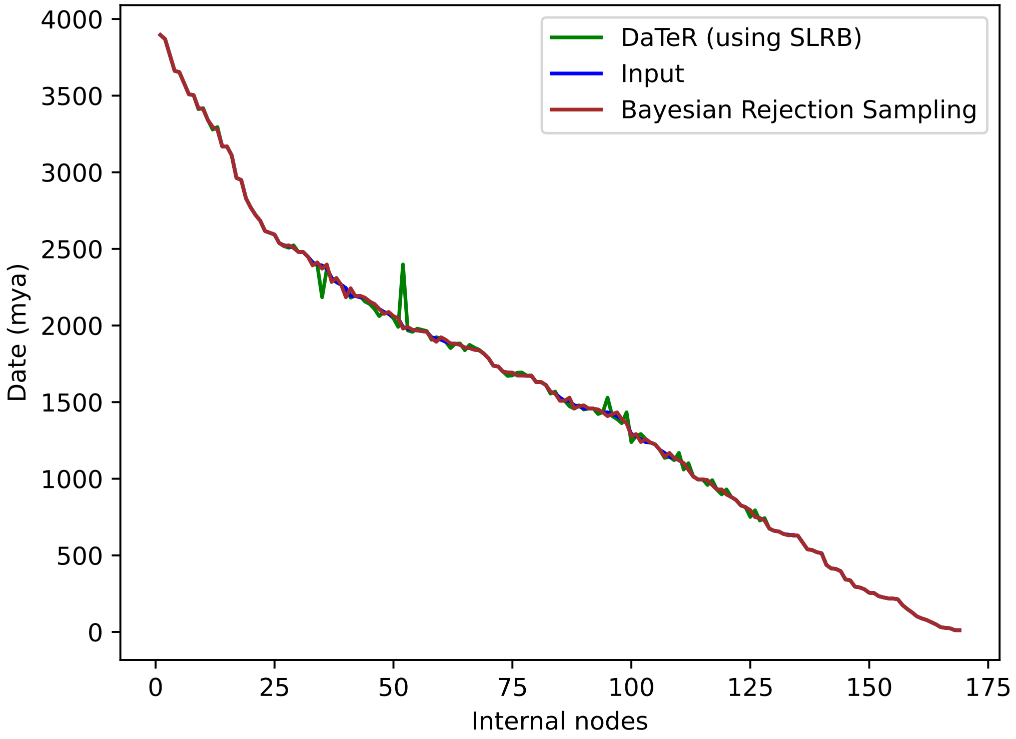

Figure S1: **Differences in assigned node dates for CIR_nobd.** Node dates assigned in the aggregated input chronograms, DaTeR-corrected (using SLRB) chronograms, and Bayesian rejection-sampling chronograms for the CIR_nobd molecular dating model are shown. Nodes are ordered by decreasing order of dates in the input chronogram. Dates are in units of mya.

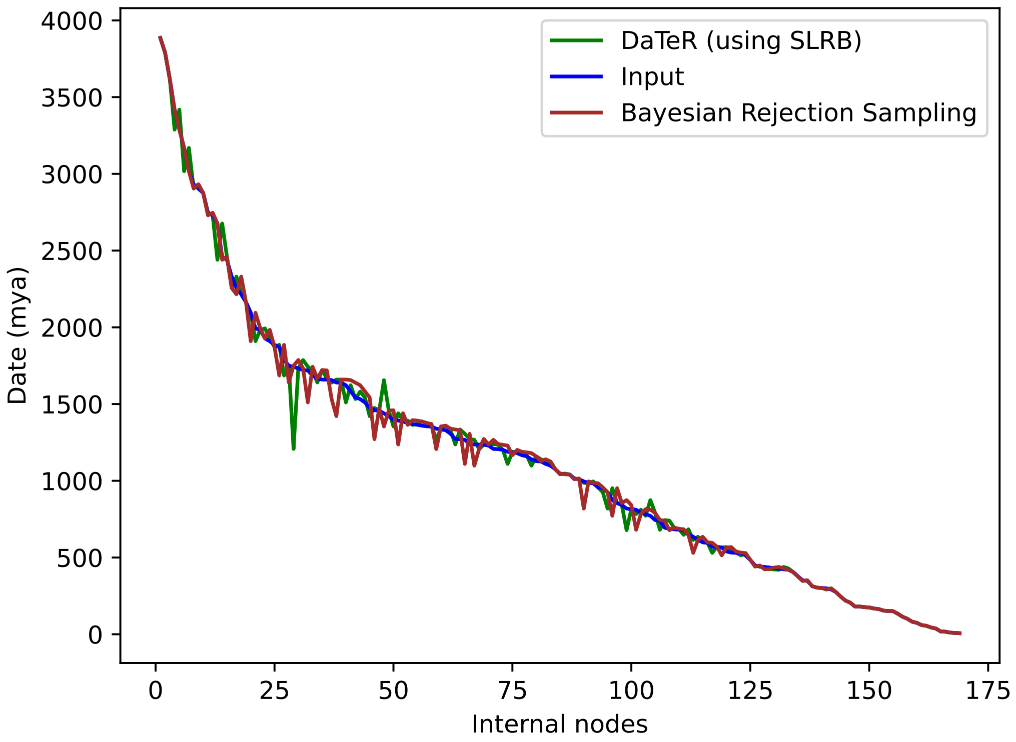

Figure S2: **Differences in assigned node dates for CIR_bd.** Node dates assigned in the aggregated input chronograms, DaTeR-corrected (using SLRB) chronograms, and Bayesian rejection-sampling chronograms for the CIR_bd molecular dating model are shown. Nodes are ordered by decreasing order of dates in the input chronogram. Dates are in units of mya.

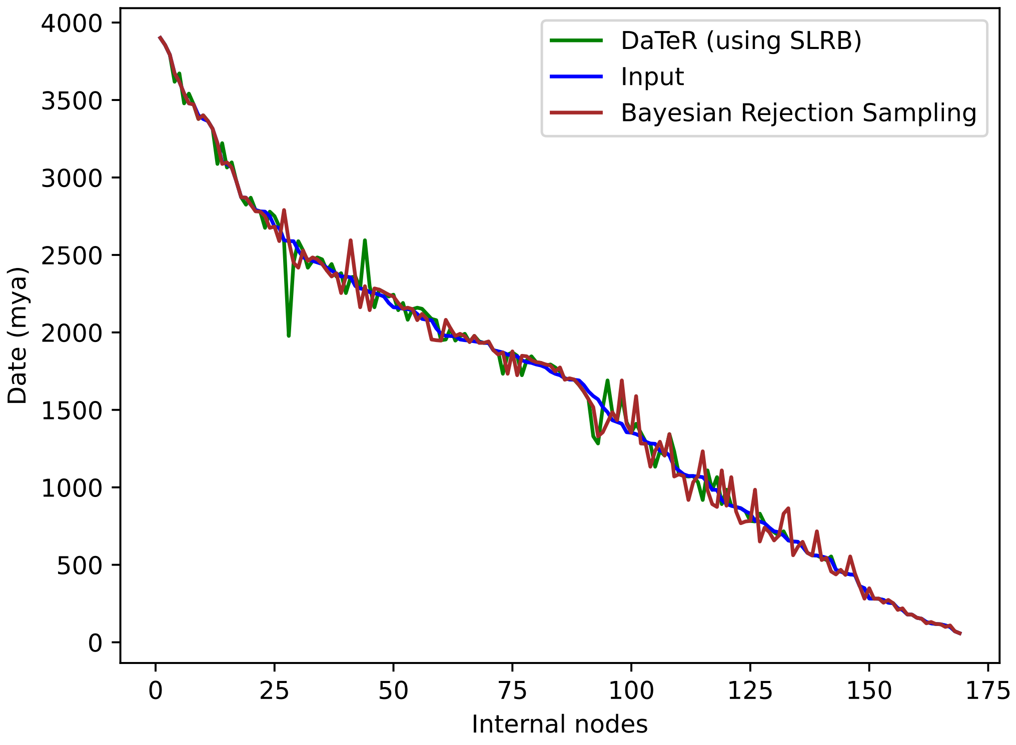

Figure S3: **Differences in assigned node dates for LN_nobd.** Node dates assigned in the aggregated input chronograms, DaTeR-corrected (using SLRB) chronograms, and Bayesian rejection-sampling chronograms for the LN_nobd molecular dating model are shown. Nodes are ordered by decreasing order of dates in the input chronogram. Dates are in units of mya.

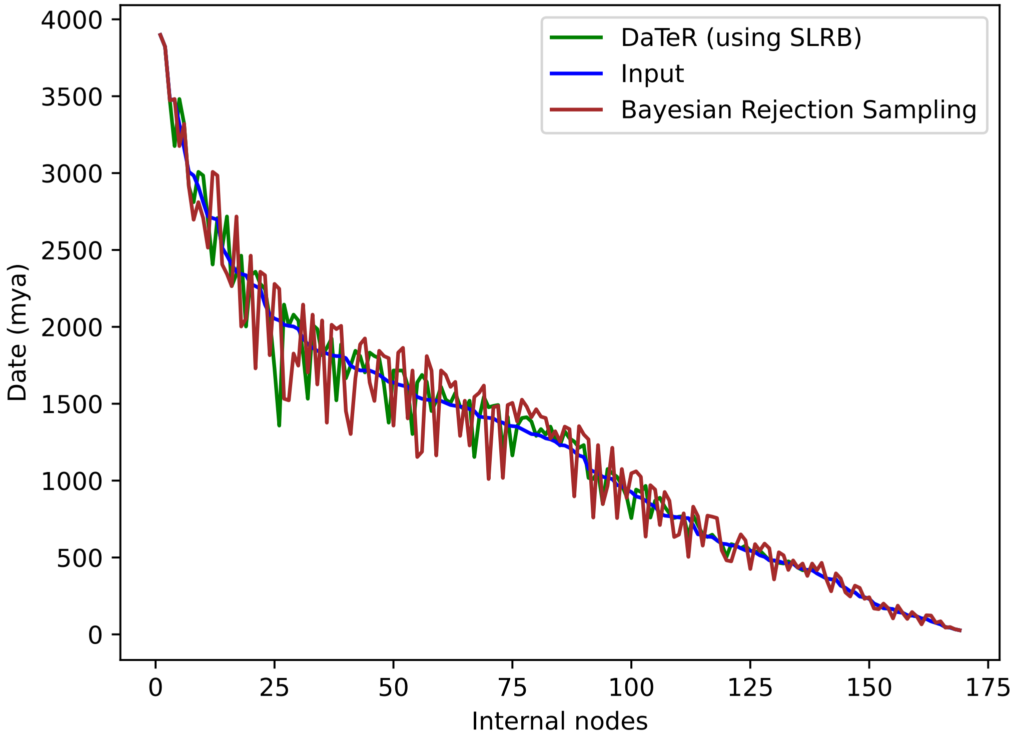

Figure S4: **Differences in assigned node dates for LN_bd.** Node dates assigned in the aggregated input chronograms, DaTeR-corrected (using SLRB) chronograms, and Bayesian rejection-sampling chronograms for the LN_bd molecular dating model are shown. Nodes are ordered by decreasing order of dates in the input chronogram. Dates are in units of mya.

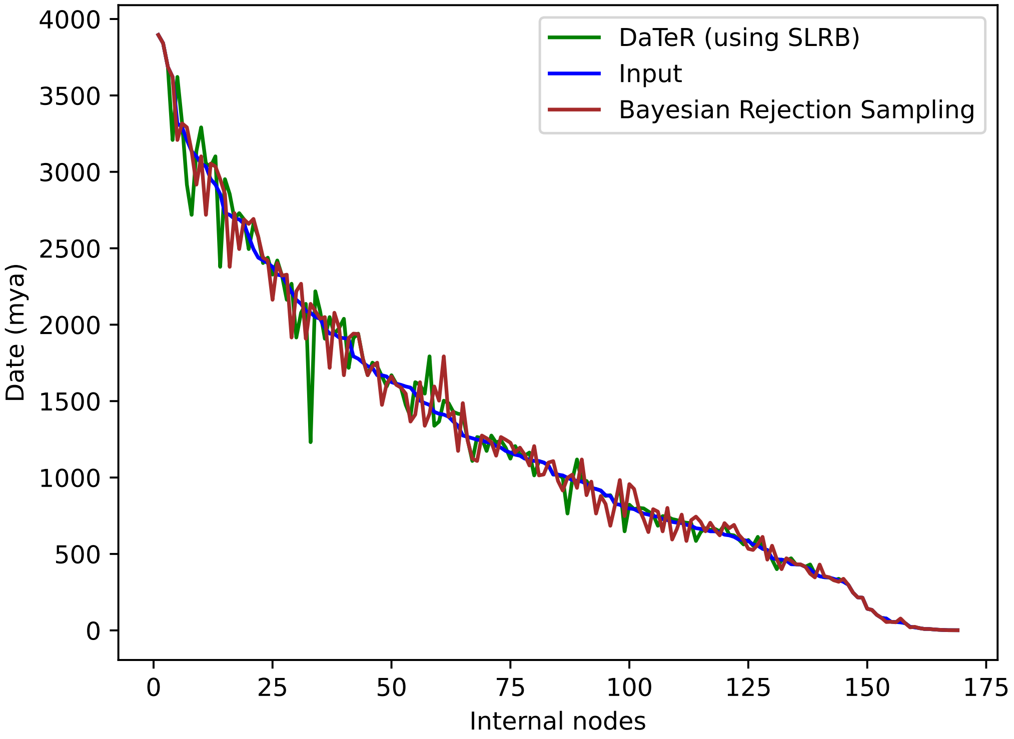

Figure S5: **Differences in assigned node dates for UGAM_nobd.** Node dates assigned in the aggregated input chronograms, DaTeR-corrected (using SLRB) chronograms, and Bayesian rejection-sampling chronograms for the UGAM_nobd molecular dating model are shown. Nodes are ordered by decreasing order of dates in the input chronogram. Dates are in units of mya.

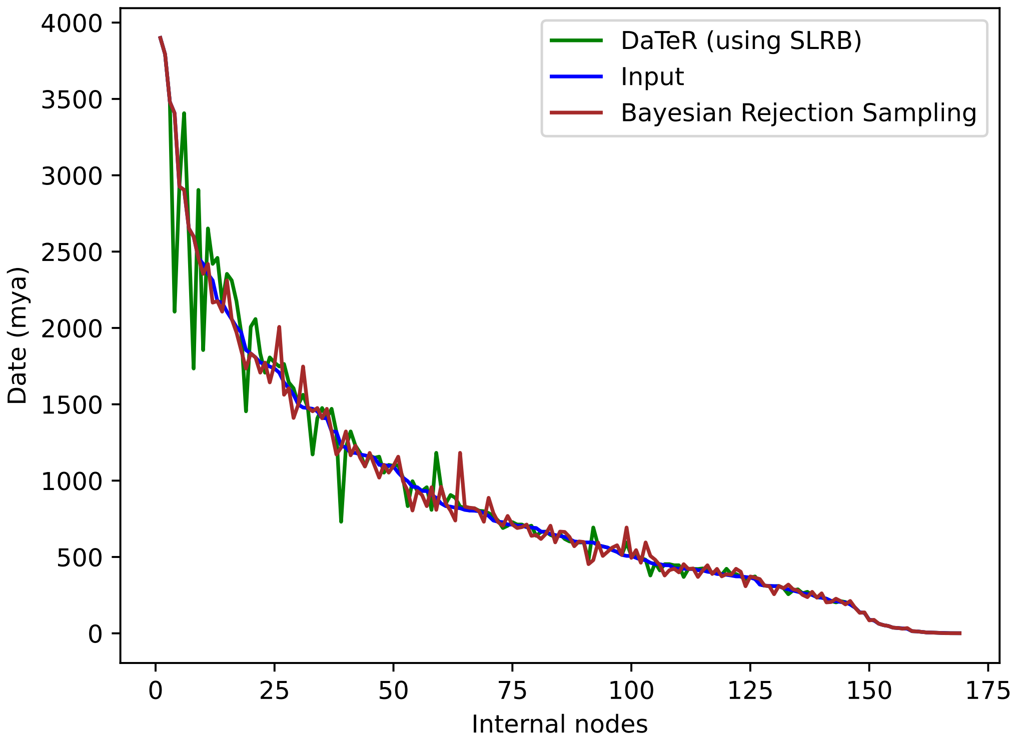

Figure S6: **Differences in assigned node dates for UGAM_bd.** Node dates assigned in the aggregated input chronograms, DaTeR-corrected (using SLRB) chronograms, and Bayesian rejection-sampling chronograms for the UGAM_bd molecular dating model are shown. Nodes are ordered by decreasing order of dates in the input chronogram. Dates are in units of mya.

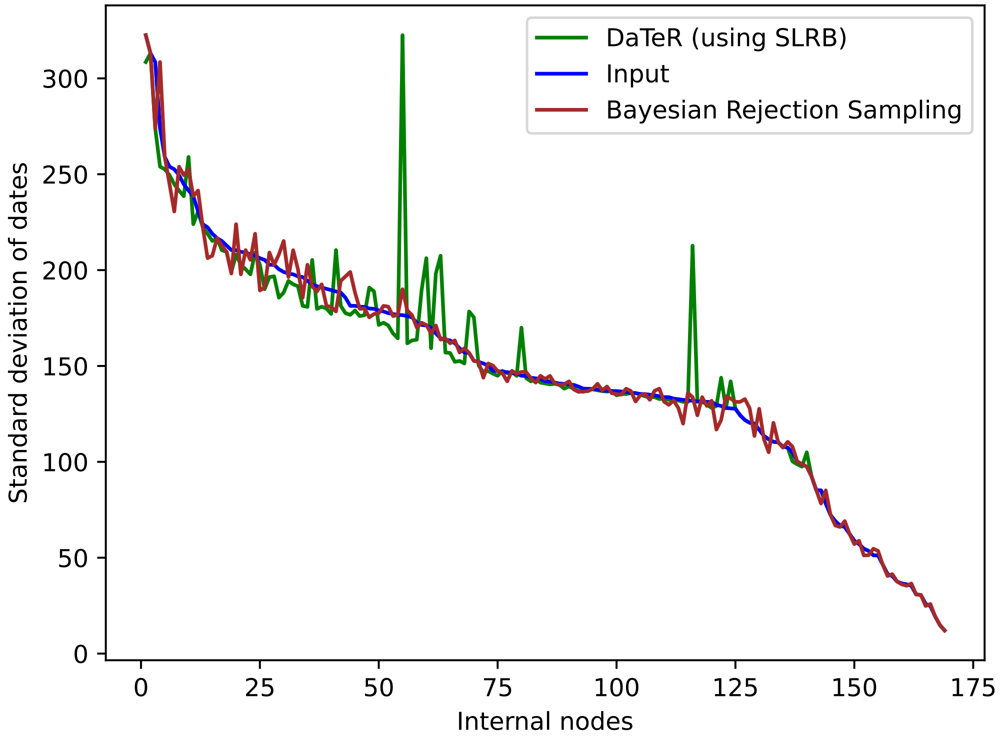

Figure S7: **Differences in standard deviations of assigned dates for CIR_nobd.** Standard deviations for node dates assigned in the 1000 input chronograms, 1000 DaTeR-corrected (using SLRB) chronograms, and selected Bayesian rejection-sampling chronograms for the CIR_nobd molecular dating model are shown. Nodes are ordered by decreasing order of date standard deviations in the input chronogram. All standard deviations are in units of million years.

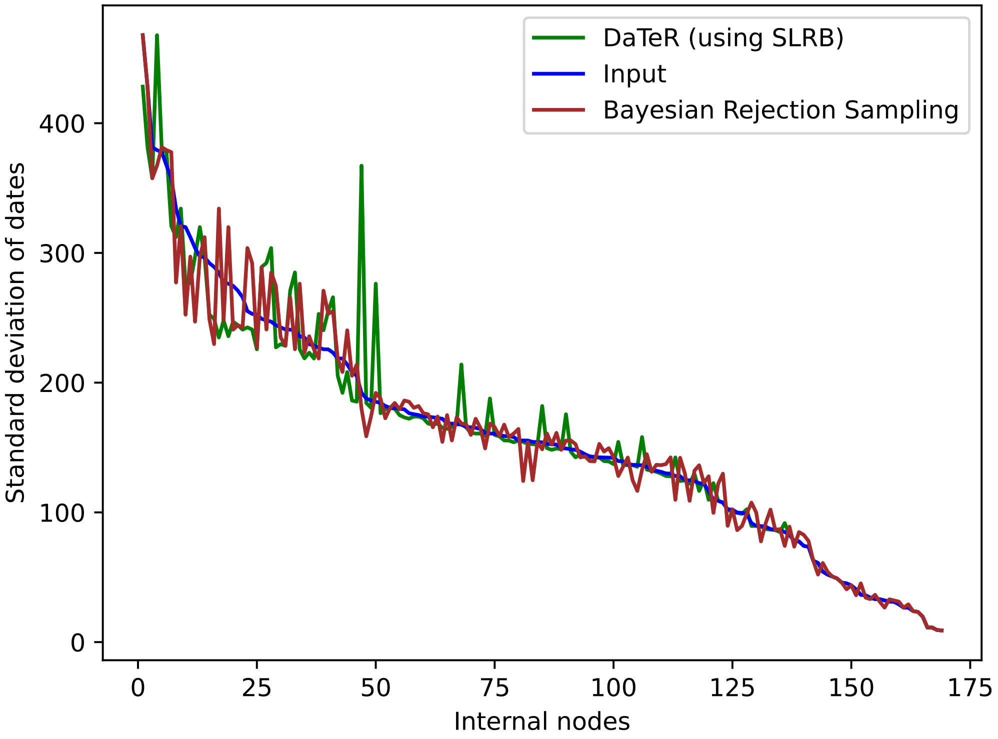

Figure S8: **Differences in standard deviations of assigned dates for CIR_bd.** Standard deviations for node dates assigned in the 1000 input chronograms, 1000 DaTeR-corrected (using SLRB) chronograms, and selected Bayesian rejection-sampling chronograms for the CIR_bd molecular dating model are shown. Nodes are ordered by decreasing order of date standard deviations in the input chronogram. All standard deviations are in units of million years.

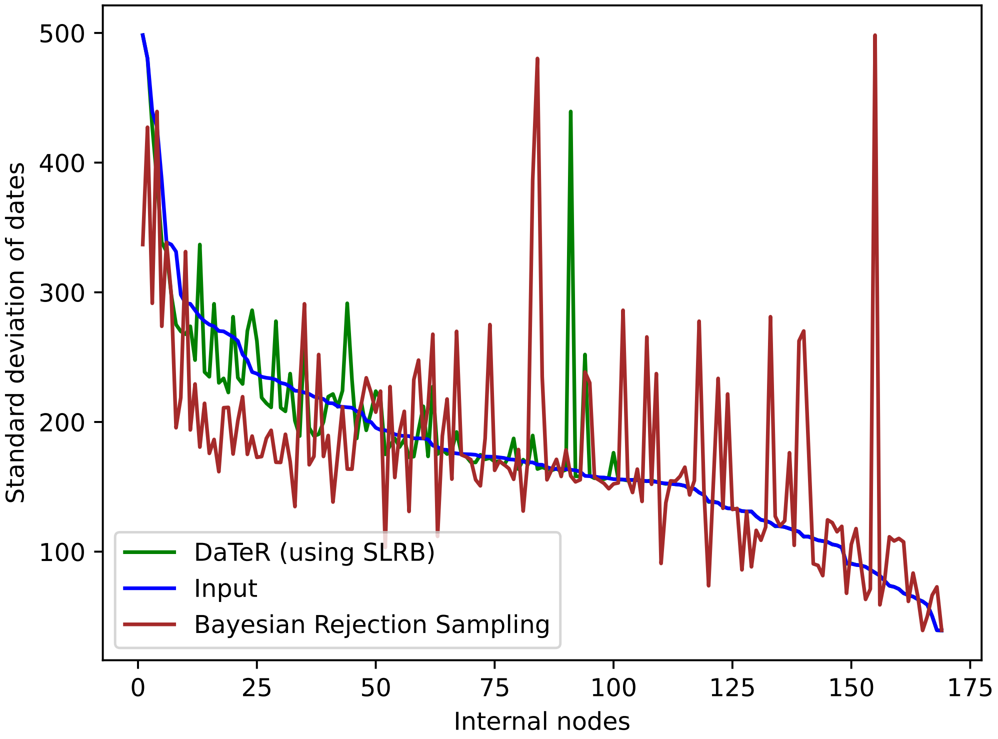

Figure S9: **Differences in standard deviations of assigned dates for LN_nobd.** Standard deviations for node dates assigned in the 1000 input chronograms, 1000 DaTeR-corrected (using SLRB) chronograms, and selected Bayesian rejection-sampling chronograms for the LN_nobd molecular dating model are shown. Nodes are ordered by decreasing order of date standard deviations in the input chronogram. All standard deviations are in units of million years.

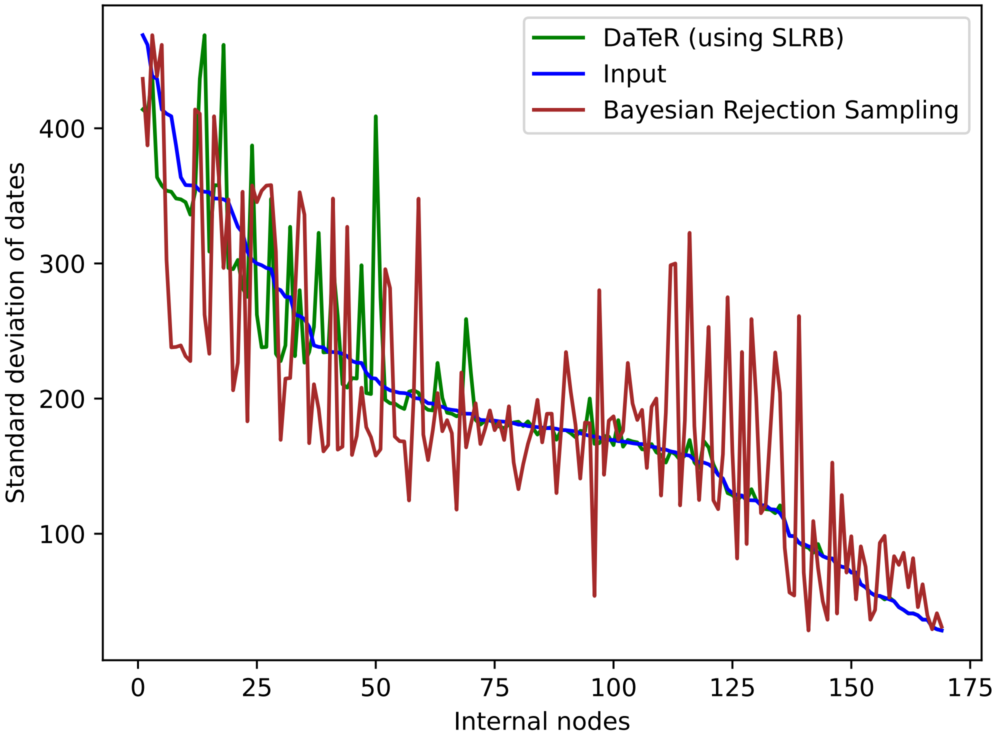

Figure S10: **Differences in standard deviations of assigned dates for LN_bd.** Standard deviations for node dates assigned in the 1000 input chronograms, 1000 DaTeR-corrected (using SLRB) chronograms, and selected Bayesian rejection-sampling chronograms for the LN_bd molecular dating model are shown. Nodes are ordered by decreasing order of date standard deviations in the input chronogram. All standard deviations are in units of million years.

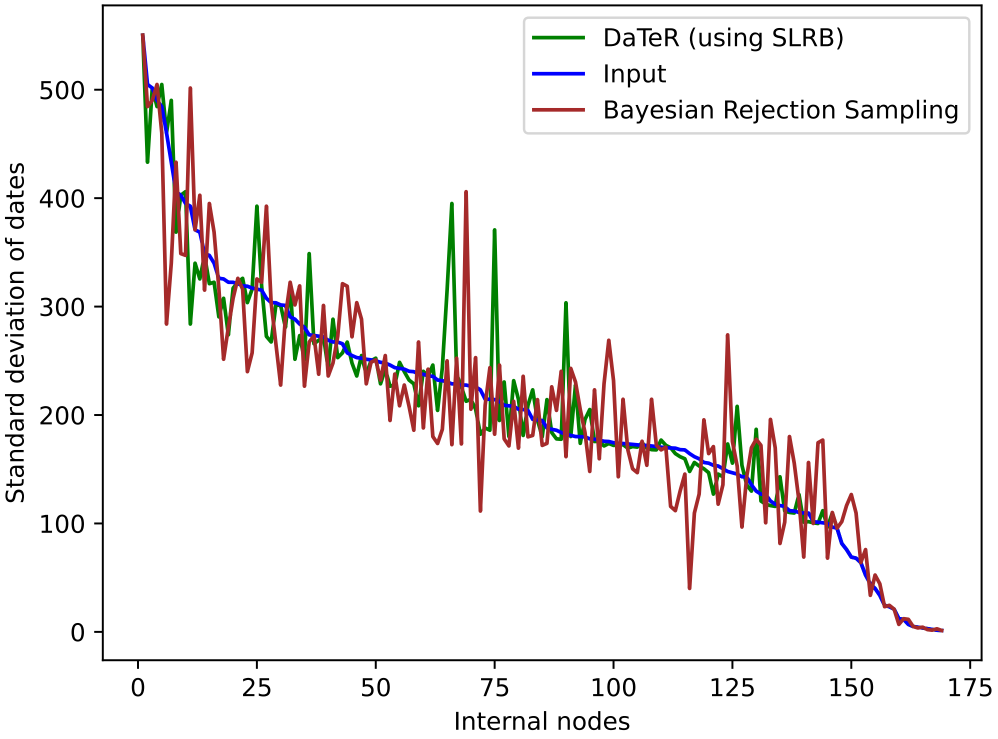

Figure S11: **Differences in standard deviations of assigned dates for UGAM_nobd.** Standard deviations for node dates assigned in the 1000 input chronograms, 1000 DaTeR-corrected (using SLRB) chronograms, and selected Bayesian rejection-sampling chronograms for the UGAM_nobd molecular dating model are shown. Nodes are ordered by decreasing order of date standard deviations in the input chronogram. All standard deviations are in units of million years.

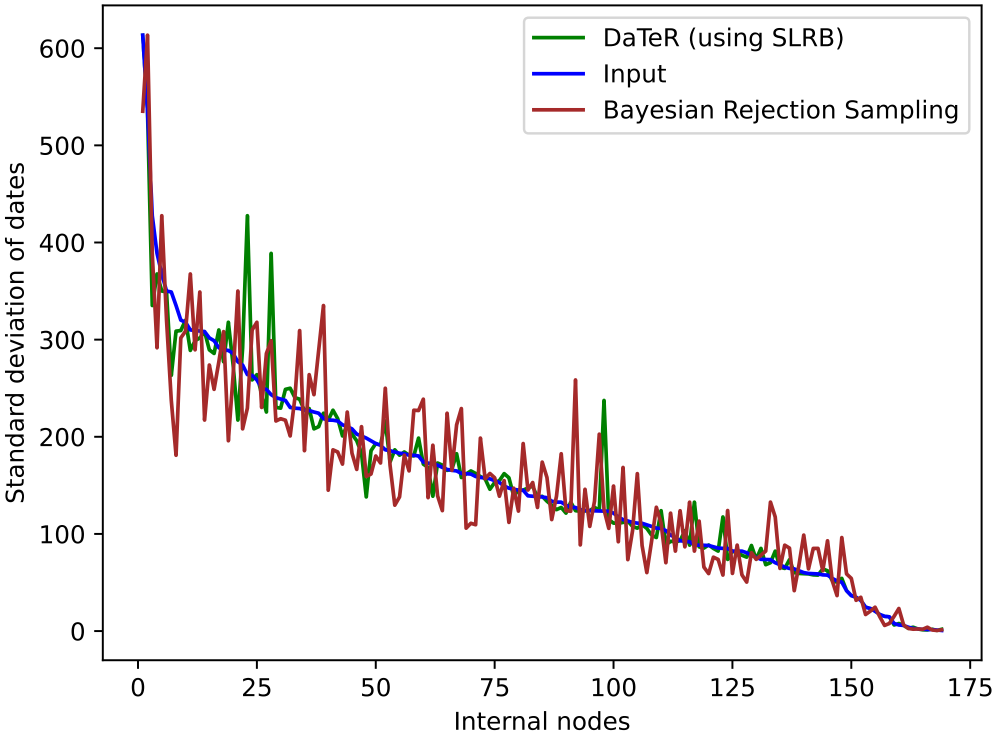

Figure S12: **Differences in standard deviations of assigned dates for UGAM_bd.** Standard deviations for node dates assigned in the 1000 input chronograms, 1000 DaTeR-corrected (using SLRB) chronograms, and selected Bayesian rejection-sampling chronograms for the UGAM_bd molecular dating model are shown. Nodes are ordered by decreasing order of date standard deviations in the input chronogram. All standard deviations are in units of million years.

References

A. J. Drummond, S. Y. W. Ho, M. J. Phillips, and A. Rambaut. Relaxed phylogenetics and dating with confidence. *PLoS biology*, 4(5):e88, 2006.

G. Fournier, K. Moore, L. Rangel, J. Payette, L. Momper, and T. Bosak. The archean origin of oxygenic photosynthesis and extant cyanobacterial lineages. *Proceedings of the Royal Society B*, 288(1959):20210675, 2021.

J. Huerta-Cepas, D. Szklarczyk, D. Heller, A. Hernandez-Plaza, S. K. Forslund, H. Cook, D. R. Mende, I. Letunic, T. Rattei, L. J. Jensen, C. von Mering, and P. Bork. eggNOG 5.0: a hierarchical, functionally and phylogenetically annotated orthology resource based on 5090 organisms and 2502 viruses. *Nucleic Acids Research*, 47(D1):D309–D314, 11 2018. ISSN 0305-1048. doi: 10.1093/nar/gky1085. URL `https://doi.org/10.1093/nar/gky1085`.

S. Kundu and M. S. Bansal. SaGePhy: an improved phylogenetic simulation framework for gene and subgene evolution. *Bioinformatics*, 35(18):3496–3498, 02 2019. doi: 10.1093/bioinformatics/btz081. URL `https://doi.org/10.1093/bioinformatics/btz081`.

N. Lartillot and H. Philippe. A bayesian mixture model for across-site heterogeneities in the amino-acid replacement process. *Mol. Biol. Evol.*, 21(6):1095–1109, 2004.

T. Lepage, D. Bryant, H. Philippe, and N. Lartillot. A General Comparison of Relaxed Molecular Clock Models. *Mol. Biol. Evol.*, 24(12):2669–2680, 09 2007. ISSN 0737-4038. doi: 10.1093/molbev/msm193. URL `https://doi.org/10.1093/molbev/msm193`.

L.-T. Nguyen, H. A. Schmidt, A. von Haeseler, and B. Q. Minh. IQ-TREE: A Fast and Effective Stochastic Algorithm for Estimating Maximum-Likelihood Phylogenies. *Molecular Biology and Evolution*, 32(1):268–274, 11 2014. ISSN 0737-4038. doi: 10.1093/molbev/msu300. URL `https://doi.org/10.1093/molbev/msu300`.

B. Rannala and Z. Yang. Inferring Speciation Times under an Episodic Molecular Clock. *Systematic Biology*, 56(3):453–466, 06 2007. ISSN 1063-5157. doi: 10.1080/10635150701420643. URL `https://dx.doi.org/10.1080/10635150701420643`.

J. L. Thorne, H. Kishino, and I. S. Painter. Estimating the rate of evolution of the rate of molecular evolution. *Mol. Biol. Evol.*, 15(12):1647–1657, 1998.
